# A Fatal Case Report of Chenopodium ambrosioides L. (M’khinza) Intoxication

**DOI:** 10.7759/cureus.60351

**Published:** 2024-05-15

**Authors:** Hasnae Elhaddadi, Amal Hamami, Aziza Elouali, Abdeladim Babakhouya, Maria Rkain

**Affiliations:** 1 Department of Pediatrics, University Hospital Mohammed VI, Faculty of Medicine and Pharmacy, Mohammed I University, Oujda, MAR

**Keywords:** child, multi-visceral failure, phytotherapy, intoxication, chenopodium ambrosioides l

## Abstract

Chenopodium ambrosioides L. is a plant belonging to the Chenopodiaceae family, known for its use in traditional medicine as a remedy for its antiseptic, analgesic, antipyretic, antispasmodic, and anti-inflammatory properties. It is used for its therapeutic properties internally as an infusion or externally as a vermifuge. Its use can be responsible for serious, even fatal, side effects and intoxications, particularly in infants and children. These may include neurological, digestive, hepatic, or renal complications. We present a case of Chenopodium ambrosioides L. intoxication in a four-year-old girl, resulting from repeated high-dose infusions of this plant for antipyretic purposes. She was admitted to the pediatric emergency department for management of a disorder of consciousness four hours after ingesting Chenopodium ambrosioides L. to treat acute fever.

## Introduction

Chenopodium ambrosioides L., also known as ansérine, or M’khinza in the Moroccan dialect, is a plant belonging to the Chenopodiaceae family known for its use in traditional Moroccan medicine as a remedy for its antiseptic, analgesic, antipyretic, antispasmodic and anti-inflammatory properties. Several studies have demonstrated the effectiveness of this plant in therapeutic use, but the therapeutic range remains very limited [1]. Therapeutic use by infusion or cutaneous application may be responsible for serious or even fatal adverse effects and intoxications, especially in infants and children. These may include neurological, digestive, hepatic, or renal complications [2]. We report a case of Chenopodium ambrosioides L. intoxication in a four-year-old girl, resulting from repeated high-dose infusions of this plant for antipyretic purposes.

## Case presentation

A four-year-old patient from a non-consanguineous marriage and the only daughter of her parents was admitted to the pediatric emergency department for management of a consciousness disorder. The mother reported the notion of applying M'khinza all over the body as a cataplasm, and administering 4 cups orally as an infusion of the same plant, used as an antipyretic to treat an acute fever with no associated clinical signs that had developed less than 24 hours before her admission. Four hours after ingesting M'khinza, the patient presented with profuse diarrhea associated with incoercible vomiting and consciousness disorders.

Upon clinical examination, the patient was drowsy with a Glasgow score of 12/15, dehydrated stage B, tachycardic at 132 beats/minute, polypneic at 31 cycles/minute, oxygen saturation was 98%, blood pressure was 08/03 cmHg, capillary blood glucose was 0.87 g/l, and temperature was 36.9◦C. Abdominal examination showed increased peristalsis. Pleuropulmonary auscultation did not objectify any rales. Cardiovascular and skin examinations were without abnormalities. The rest of the somatic examination was normal. After conditioning, the biological workup revealed hepatic cytolysis and cholestasis, renal failure, hyponatremia, and hypocalcemia. Toxicological tests were not performed (Table 1).

**Table 1:** Our patient's biological findings upon admission SGOT: serum glutamic oxaloacelic transminases; SGPT: serum glutamic pyruvic transaminases; GGT: gamma-glutamyl-transferase; ALP: alkaline phosphatase

Laboratory parameter	values	Reference range
White blood cell (/μg)	5740	4000 – 10 000
Lymphocytes (/μg)	2150	2000 – 4000
Neutrophil (/μg)	3350	1500 – 7000
Hemoglobin (g/dl)	13.2	12 - 16
Platelets (/μg)	180 000	150 000 – 450 000
SGOT (UI/l)	476	5 – 35
SGPT (UI/l)	188	5 – 55
GGT (UI/l)	102	9 – 36
ALP (UI/l)	172	40 – 150
Sodium mEq/l	124	138 – 145
Potassium mEq/l	3.6	3.4 – 4.7
Calcium mg/l	79	88 – 108
Blood urea g/l	1.29	0.10 – 0.30
Blood creatinine mg/l	16.20	3.1 – 4.7
Prothrombin time %	78	70 – 100

Because of her neurological, hemodynamic, and respiratory instability, the patient was admitted to the pediatric intensive care unit for further management. She was intubated, ventilated, sedated, and put on vasoactive drugs and broad-spectrum antibiotics. After admission to intensive care, the outcome was fatal. During transfer to the radiology unit for MRI brain imaging, the patient presented a cardiopulmonary arrest, which did not recover after cardiopulmonary resuscitation measures.

## Discussion

Despite advances in pharmacology, the use of medicinal plants has increased in most of the world, especially in developing countries. This may be due to the idea that plants are a natural, risk-free means of treatment [3]. Morocco, with its rich and diverse flora, constitutes a phytogenetic reservoir with some 4,500 plant species and subspecies. As a result, plant intoxication is a major public health problem. It can be accidental or deliberate, and it can be lethal [4]. Previous studies by the Moroccan Poison Control Centre have shown that plants are responsible for 3 to 5% of all intoxications, sometimes with a high lethality rate (17%), while death from Dysphania ambrosioides was ranked fourth (6%) in the distribution of cases of death by plants and traditional pharmacopeia products between 2009 and 2020 [1].

In the literature, data on anserine intoxication in children are rare and poorly documented, but research on its uses, compositions, therapeutic effects, and phytotoxicity is extensive [3]. Chenopodium ambrosioides L. has long been used in traditional medicine, as it is highly recommended by herbalists as an antiseptic, analgesic, antipyretic, antispasmodic, anti-inflammatory, galactogenic, or for its healing effect in oral aphthosis and skin wounds. All parts of the plant (root, leaves, flowers, seeds), whether fresh or dried, are used [5].

The most important active ingredients in the essential oil of Chenopodium ambrosioides leaves are p-cymene (50.0%), alpha-terpinene (37.6%), and ascaridol (3.5%) [6]. According to the Moroccan Poison Control Center, the clinical presentation of Chenopodium ambrosioides L. intoxication in children can manifest as hepatic, renal, neurological, digestive, cutaneous damage, altered general condition, and multi-visceral failure [1]. Several studies have reported cases of liver toxicity following anserine consumption. Cases of hepatic cytolysis and cholestasis have been described in all studies [5,7].

In the literature, renal toxicity to Chenopodium ambrosioides can result in tubular necrosis and glomerular damage, with infiltration of the interstitium by inflammatory cells. Renal biopsy confirms acute tubulointerstitial nephritis. These effects have been observed following chronic ingestion of large doses [8]. Cases of toxic encephalopathy following ingestion of Chenopodium ambrosioides are rarely described in the literature. Chaoui et al. reported a case of toxic encephalopathy in a six-month-old infant, with fatal evolution. The mechanism of anserine neurotoxicity is unclear. Its toxic effects are rarely reported in the literature [9].

In the case of our patient, following a decision to increase the dose and multiply the doses when the desired effect was not achieved, she showed signs of neurological and gastrointestinal toxicity respectively, leading to her death in less than six hours.

## Conclusions

The cases of neurotoxicity, enterotoxicity, and renal toxicity in M’khinza call attention to the need to know how to evoke the diagnosis of Chenopodium ambrosioides L. intoxication, to inform and fight against the banalization of its consumption, and to encourage research into traditional pharmacology to identify its therapeutic properties to rationalize and codify its use.
